# Comprehensive Identification of Protein Substrates of the Dot/Icm Type IV Transporter of *Legionella pneumophila*

**DOI:** 10.1371/journal.pone.0017638

**Published:** 2011-03-09

**Authors:** Wenhan Zhu, Simran Banga, Yunhao Tan, Cheng Zheng, Robert Stephenson, Jonathan Gately, Zhao-Qing Luo

**Affiliations:** 1 Department of Biological Sciences, Purdue University, West Lafayette, Indiana, United States of America; 2 Department of Statistics, Purdue University, West Lafayette, Indiana, United States of America; University of Louisville, United States of America

## Abstract

A large number of proteins transferred by the *Legionella pneumophila* Dot/Icm system have been identified by various strategies. With no exceptions, these strategies are based on one or more characteristics associated with the tested proteins. Given the high level of diversity exhibited by the identified proteins, it is possible that some substrates have been missed in these screenings. In this study, we took a systematic method to survey the *L. pneumophila* genome by testing hypothetical *orfs* larger than 300 base pairs for Dot/Icm-dependent translocation. 798 of the 832 analyzed *orfs* were successfully fused to the carboxyl end of β-lactamase. The transfer of the fusions into mammalian cells was determined using the β-lactamase reporter substrate CCF4-AM. These efforts led to the identification of 164 proteins positive in translocation. Among these, 70 proteins are novel substrates of the Dot/Icm system. These results brought the total number of experimentally confirmed Dot/Icm substrates to 275. Sequence analysis of the C-termini of these identified proteins revealed that Lpg2844, which contains few features known to be important for Dot/Icm-dependent protein transfer can be translocated at a high efficiency. Thus, our efforts have identified a large number of novel substrates of the Dot/Icm system and have revealed the diverse features recognizable by this protein transporter.

## Introduction

*Legionella pneumophila* is an opportunistic bacterial pathogen, which is ubiquitous in the environment as a parasite of fresh water amoebae. Inhalation of *L. pneumophila* contaminated aerosols by immuno-compromised individuals can lead to an atypical acute pneumonia known as Legionnaires' disease [1]. The cell biological features associated with infections of amoeba and mammalian cells are highly similar, suggesting that amoeba serves as the “training site” for its ability to colonize higher organisms [2]. Similarly, most genetic determinants important for multiplying within amoebae cells also are essential for its growth in mammalian cells [3]. The single most important virulence factor of *L. pneumophila* is the Dot/Icm type IV secretion system [4], [5]. Built with about 26 proteins, this apparatus connects the bacterial cytoplasm to the extracellular environment and functions as the conduit through which effector proteins are delivered into host cells [6].

Protein substrates of the Dot/Icm are directly involved in the construction of the niche called Legionella containing vacuole (LCV) that supports intracellular bacterial growth [7]. Elucidating the functions of these substrates will reveal not only the mechanisms used by *L. pneumophila* to subvert host cellular processes but also could potentially reveal novel host pathways undetectable or difficult to study under normal physiological conditions. Thus, tremendous efforts have been invested to identify such effector proteins, characterize their functions, and to understand their roles in *L. pneumophila* pathogenesis [8]. Several methods have been developed to measure Dot/Icm-mediated protein translocation: i) immunostaining of infected cells or/and isolated LCVs with antibodies specific for candidate effector proteins [9], [10]; ii) interbacterial protein transfer by detecting in recipient cells the deletion of genes by chimeras of candidate proteins and the Cre recombinase [10]; iii) the restoration of a transfer-deficient mutant of the effector SidC [11], [12]; iv) the use of the calmodulin-dependent adenylate cyclase from *Bordetella pertussis* as a reporter [13], [14], [15], [16] and v), a FRET assay based on β-lactamase activity and the reporter reagent CCF4-AM [17]. Candidate genes used in these translocation assays were obtained by a number of strategies, including bioinformatics analyses to retrieve proteins harboring structural features or functional domains typically found in proteins of eukaryotes origins [18]; second, proteins that physically interact with components of the Dot/Icm complex or chaperones [10], [14]; third, proteins capable of disrupting cellular processes of *Saccharomyces cerevisiae* [19], [20], [21]; fourth, proteins whose expression appears to be regulated similarly to known substrates [16], [22], [23]; fifth, computational tools to search for proteins which have one or more of the above features [24]. The combination of these gene search methods and the use of one or more translocation reporter systems have led to the identification of 204 proteins transferred by Dot/Icm.

A hydrophobic residue at the -3^rd^ position [15] and the E-block [12] are the two known features important for Dot/Icm-dependent translocation of subsets of substrates. Other characteristics, such as frequent occurrence of small side-chain residues at -11th to -5th residues, and a polar residue at the -16^th^ position had been found in many substrates [25]. Whether these features are important for protein translocation is unknown. Dot/Icm substrates identified from candidates that have not been prescreened will likely lead to reveal novel features recognizable by the transporter.

Consistent with early observations that the LCVs are closely associated with the rough endoplasmic reticulum (ER) of host cell and that disruption of the vesicle budding from the ER repressed intracellular bacterial replication [26], [27], several bacterial proteins have been shown to target molecules that regulate protein trafficking between the ER and the Golgi apparatus. For example, RalF activates the small GTPase Arf1 on the surface of LCVs [9] whereas SidM/DrrA and LepB are guanine nucleotide exchange factor (GEF) and GTPase activation protein (GAP) for the Ras-family small GTPase Rab1, respectively [28], [29].

In addition to vesicle trafficking, several other cellular processes are modulated by substrates of the Dot/Icm system. Mammalian cells harboring replicating *L. pneumophila* exhibits a strong resistance to exogenous cell death stimuli [30], [31], probably by synergized effects resulted from NF-κB activation and the activities of some effectors such as SdhA and SidF, which have been shown to contribute to such resistance by different mechanisms [32], [33]. Two effectors, LegK1 and LnaB are able to activate NF-κB when ectopically expressed in mammalian cells, but whether such activation plays any role in modulating host cell death is unknown [34], [35]. Like other intravacuolar pathogens, *L. pneumophila* is able to maintain a neutral luminal pH in LCVs [36]. Recently, the effector SidK has been shown to contribute to such regulation by antagonizing the activity of v-ATPase, the proton transfer machinery that controls organellar pH [20]. The cytoplasmic face of LCVs is decorated by ubiquitinated proteins and the host proteasome function is important for intracellular bacterial replication [37]. Not surprisingly, a number of Dot/Icm substrates are involved in protein ubiquitination [25], [38], [39]. Finally, at least four proteins are capable of inhibiting host protein synthesis by inactivating the translation elongation factor eEF1A, which may contribute to the induction of host stress response and other unknown cellular processes [21], [40], [41].

Given the large number of substrates, the diverse host functions modulated by these proteins and the possibility that translocation signals may be one of the parameters that control temporal translocation of these proteins, it is likely that previous screens have missed some substrates with undefined features. In this study we have greatly expanded the repertoire of Dot/Icm substrates by performing a comprehensive screen to test for translocation all the open reading frames larger than 300 base pairs annotated as hypothetical proteins in the genome *L. pneumophila* strain Philadelphia 1. Our efforts led to the identification of 70 novel Dot/Icm substrates. Furthermore, a protein translocated at a high efficiency does not share most of the features found in one or more groups of established substrates, indicating that the Dot/Icm system has a great flexibility in recognizing substrates.

## Results

### Construction of a library expressing β-lactamase and Legionella proteins fusions

In order to obtain a more complete list of protein substrates transferred by the *L. pneumophila* Dot/Icm system, we initiated a comprehensive screen to test Dot/Icm-dependent translocation of hypothetical proteins in strain Philadelphia 1. We chose the β-lactamase as the reporter because this system is applicable for large-scale screens while holding comparable sensitivity to other systems such as the adenylate cyclase (Cya) assay [17]. In this method, each candidate gene is fused to TEM1 (β-lactamase) and the bacterial strain expressing the fusion protein is used to infect host cells. Host cells are then loaded with CCF4-AM which, when excited at 409 nm, emits green fluorescence (520 nm) due to fluorescence resonance energy transfer (FRET) between the coumarin and fluorescein fluorophores. Delivery of the β-lactamase fusion protein into host cells leads to cleavage of the β-lactam ring of CCF4-AM, releasing the two fluorophores and changing the fluorescence emission from green to blue (447 nm) when excited at the same wavelength. Translocation detected by this reporter can be easily quantitated by the percentage of infected cells emitting blue fluorescence signals [17].

**Figure 1:**
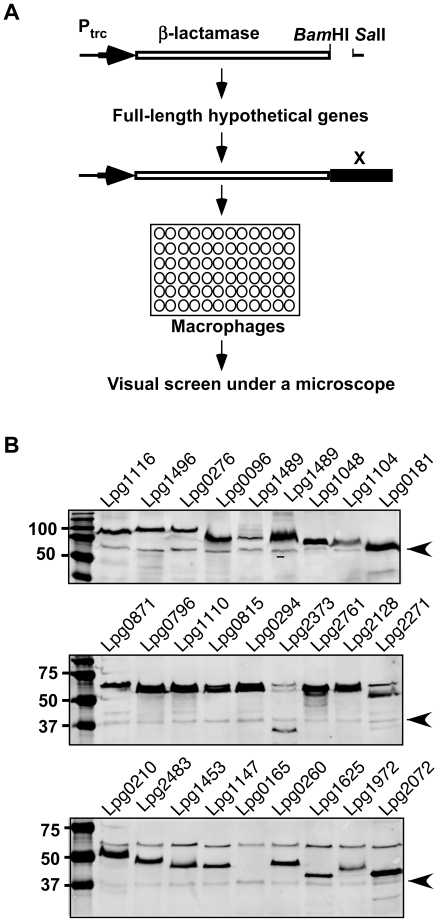
Construction of a library expressing fusions of β-lactamase and *L. pneumophila* hypothetical proteins. **A.** A schematic structure of the fusion proteins and the screening strategy. In most cases, the gene was fused with the β-lactamase by inserting into the vector as a *Bam*HI/*Sal*I fragment. After infection, samples were loaded with the CCF4-AM dye and were inspected under a fluorescence microscope. **B.** Evaluation of the library for expression of the fusion proteins. Plasmids directing expression of β-lactamase fusions were introduced into a wild type *L. pneumophila* strain. Total cell lysates of bacteria grown in the presence of IPTG were used to examine the steady state of the fusion proteins by immunoblot. In each image, the detection of a protein nonspecifically recognized by the antibody (arrow) was used as a loading control.

To construct the fusion library, we first retrieved all the open reading frames larger than 300 base pairs annotated as hypothetical genes from the *L. pneumophila* Philadelphia 1 genome (Table S1). After eliminating genes that have been reported as substrates of the Dot/Icm system at the time the project was initiated, a total of 833 candidate genes were obtained (Table S1). To clone the genes, we designed primer pairs to amplify each open reading frame by PCR and inserted them individually into pXDC61M to generate translational fusions with the upstream β-lactamase gene (Fig. 1A). A total of 798 plasmids expressing the β-lactamase fusion were constructed. To examine the quality of the library, we randomly chose 30 *L. pneumophila* strains harboring the plasmid to detect the expression of the fusion proteins. Although the level of expression varies, a protein corresponding to the expected sizes of the chimeras was detected in most of the samples (Fig. 1B), indicating that we have successfully constructed a library expressing β-lactamase fusions in *L. pneumophila*.

### Identification of proteins transferred by the Dot/Icm system

After verifying the expression of the fusion proteins in *L. pneumophila*, we infected U937 macrophages with *L. pneumophila* strains expressing β-lactamase fusions grown to post exponential phase. One hour after infection, cells were loaded with CCF4-AM dye and incubated for an additional 2 hours at room temperature. Translocation of the β-lactamase chimera was assessed by the presence of cells emitting blue fluorescence signals. A group of 24 bacterial strains expressing β-lactamase were used to infected host cells for each screen. In each experiment, we used the TEM-RalF and TEM1-FabI hybrid proteins as the positive and negative controls, respectively [17]. Samples were visually inspected under a fluorescence microscope and strains that gave more than 5% of blue cells were retained for further study. Under this experimental condition with a multiplicity of infection (MOI) of 20, 95% of the cells infected by wild type *L. pneumophila* strain expressing the TEM-RalF fusion emits blue fluorescence, whereas similar infections with the strain expressing TEM- FabI results in no blue cells (data not shown), which are consistent with results from an earlier study [17]. Infections were repeated at least twice for strains that gave positive translocation results. Constructs harboring genes exhibiting detectable transfer were introduced into the *dot/icm*-deficient strain Lp03 and the resultant strains were similarly tested for delivery the β-lactamase fusions into host cells. None of these fusions caused detectable translocation in this *dot/icm*-deficient strain (data not shown).

**Figure 2:**
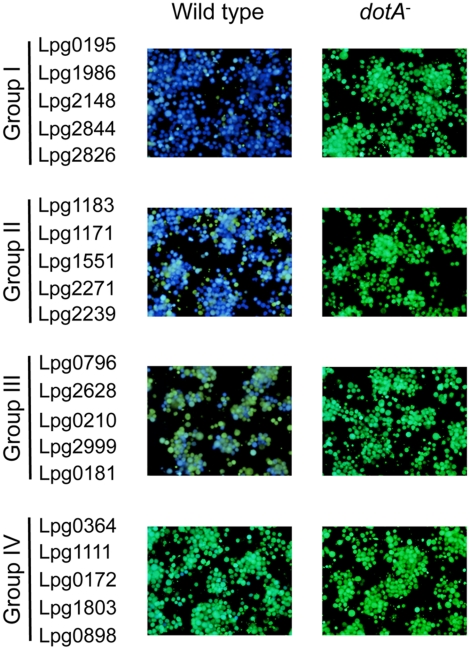
Dot/Icm-dependent translocation of substrates. Effectors identified in this study were divided into four groups according to their transfer efficiencies, 5 genes from each group were shown as representatives. U937 cells seeded in 96-well plates were infected with wild type or *dot/icm*-deficient *L. pneumophila* strains expressing a gene fusion and images were acquired 2 hrs after CCF4-AM loading with a DP72 color camera (Olympus). Group I, genes with translocation efficiency >90%; Group II, genes with translocation efficiency between 50% and 80%; Group III, genes with translocation efficiency between 20% and 45% and Group IV, genes with translocation efficiency less than 15%.

A total of 164 proteins that consistently promote TEM translational fusions in a Dot/Icm-dependent manner were obtained (Tables S2 and S3). Among these, 94 proteins had been reported as Dot/Icm substrates (Table S3), further validating the reliability of our screen strategy. Thus, our efforts have added 70 proteins to the inventory of the substrate pool of the *L. pneumophila* Dot/Icm transporter (Table S2). The transfer efficiencies of these proteins vary greatly, ranging from 5% to 95% (Fig. 2). Among these, 5 proteins exhibited transfer efficiencies comparable to that of RalF, causing more that 90% of infected cells to emit blue fluorescence signals (Fig. 2). These 5 proteins do not share any detectable common features. Instead, the primary sequence of the C-terminal portion of Lpg2844 is quite different from all known substrates (see below). Twenty-five proteins converted 50%–80% of the green cells into blue cells, 13 proteins exhibited translocation efficiencies between 20 to 45% and 27 proteins showed low transfer efficiencies with less than 20% blue cells in the samples (Fig. 2 and Table S2).

Although all candidates were annotated as hypothetical proteins in the genome database of the Philadelphia 1 strain, careful bioinformatic analysis revealed that a small fraction of them harbor motifs of known or putative functions (Table S2). Further, most of these proteins have a homolog in the genome of the Paris, Lens or the Corby strain; the number of proteins without a detectable homolog are 7, 12 and 13 for these three strains, respectively (Table S2). Two proteins, Lpg1083 and Lpg1684 are specific for the Philadelphia 1 strain (Table S2). In some cases, two or more genes were clustered in a specific region in the chromosome (Table S2), a common phenomenon in gene organization of *L. pneumophila* type IV substrates [10] and often accounts for the remarkable plasticity of genomes of this organism [42], [43].

**Figure 3:**
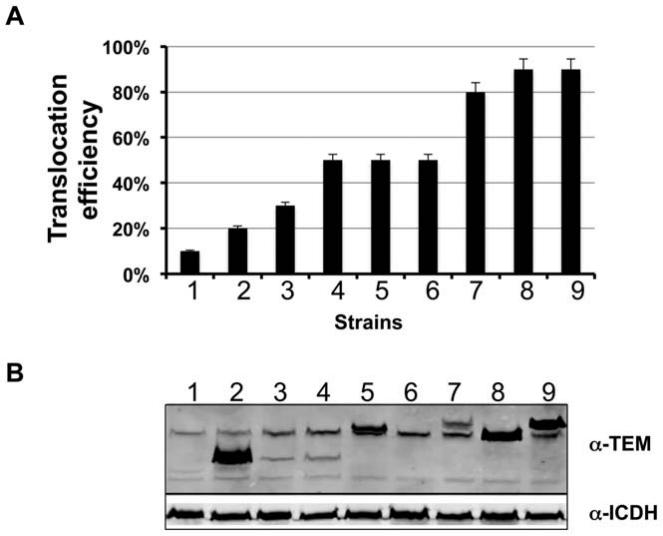
Translocation efficiency does not correlate with levels of β-lactamase fusion expressed in *L. pneumophila*. **A.** The translocation efficiency of 9 substrates in the β-lactamase assay. After CCF4-AM loading, macrophages infected with bacterial strains expression fusions between β-lactamase and individual genes were inspected under a fluorescence microscope, translocation efficiencies were obtained by enumerating cells emitting blue and green fluorescence signals, respectively. Experiments were performed in triplicates and at least 300 cells were counted each sample. Similar results were obtained in at least two independent experiments. **B.** The levels of the fusion proteins in *L. pneumophila* strains used for infections shown in A. Bacterial cells equivalent to one OD_600_ unit were lysed in 200 µl of SDS loading buffer, 15 µl of boiled supernatant were resolved by SDS-PAGE. After transferring to nitrocellulose membranes, the fusion protein was detected with a β-lactamase specific antibody by immunoblot. The isocitrate dehydrogenase (ICDH) was probed as a loading control. Samples: 1. Lpg1776; 2, Lpg0021; 3, Lpg2425; 4, Lpg1147; 5, Lpg0181; 6, Lpg2555; 7, Lpg2874; 8, Lpg0405; 9, Lpg0195.

To examine whether the difference in transfer efficiency among proteins was due to the stability of the fusion proteins, we examined the levels of the fusion proteins in several strains from each group. In general, there was no clear correlation between the steady state levels of the hybrids and translocation efficiencies. For example, the steady state levels of the TEM-Lpg0021 and TEM-Lpg0181 were among the highest in these strains, but their transfer efficiencies were not the highest (Fig. 3). On the other hand, the poorly expressed TEM-Lpg2555 and TEM-Lpg2874 were translocated at high efficiency (Fig. 3). To further determine to what extent the lack of detectable translocation was a result of a failure to express the protein fusions, we examined the β-lactamase fusions in 27 transfer deficient strains. Although the protein levels vary, all of these strains produced readily detectable fusion proteins (Fig. S1). Therefore, the level of fusion protein expressed in *L. pneumophila* is not the sole factor determining the translocation competency of a particular substrate.

### Recognition of diverse translocation signals by the Dot/Icm transporter

**Figure 4:**
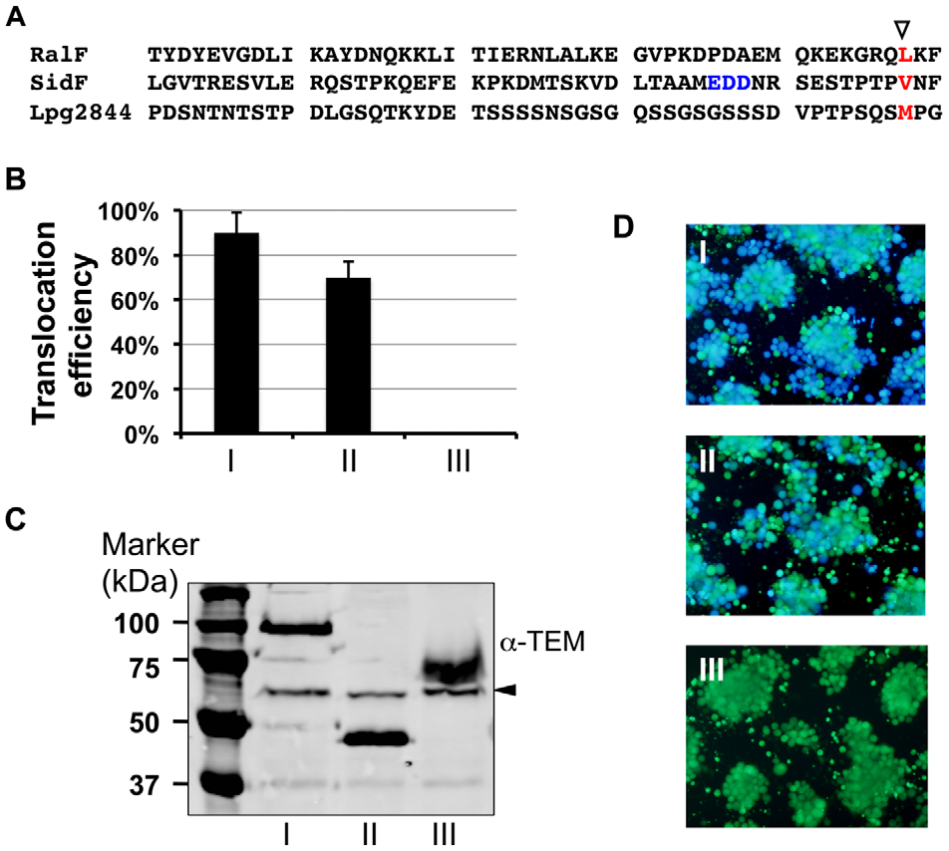
Diverse features presented in the C-terminal end of Dot/Icm substrates. **A.** Alignment of the last 50 amino acids of three well-established effectors and the new substrate Lpg2844 to highlight the features important for translocation found in Dot/Icm substrates, including: i) The hydrophobic residue at the -3^rd^ position (▽, red) [15] and ii) the E-block [12] (the three blue residues in SidF). Note the different amino acids composition in Lpg2844. **B–D.** A region containing the last 100 amino acids of Lpg2844 is important and sufficient for promoting translocation. Bacterial strains expressing fusions of β-lactamase to full-length Lpg2844 (I), it's last 100 aa (II) or a fragment lacking the last 100 aa (III) were used to infect macrophages and infected cells were loaded with the CCF4-AM dye. Translocation efficiency (B) was obtained as described in Fig. 3, data shown are the average of three independent experiments done in triplicates; stable expression of the fusions by *L. pneumophila*, equal amount of protein samples resolved by SDS-PAGE was probed for the fusions with a β-lactamase specific antibody. The ca. 60-kDa non-specific band detected by the antibody was used as a loading control (arrow in panel C). Representative images of infected cells loaded with CCF4-AM (D). Similar results were obtained in at least two independent experiments.

In our efforts to identify common features in the last 100 amino acids of the substrates that may be important for Dot/Icm-dependent protein translocation, we found that a few proteins that have amino acid composition greatly different from the known features. One such example is Lpg2844, a 361 aa protein in which more than 1/3 of the residues are serine. Interestingly, the last 100-aa region of this protein contains few of the features known to be important for Dot/Icm-dependent translocation. A hydrophobic residue (methionine) at the -3^rd^ position [15] is the only recognizable characteristics associated with translocation found on this region of this protein (Fig. 4A). Full-length Lpg2844 promoted the translocation of lactamase with 85% efficiency (Table S2 and Fig. 4 B&D). Importantly, a chimera containing β-lactamase fused to the last 100 amino acids of Lpg2844 promoted translocation at efficiencies only marginally lower than those of full-length proteins, indicating that like other Dot/Icm substrates, signals important for translocation localized to the C-terminal portion of this protein (Fig. 4B–D). As expected, a fusion that contained Lpg2844 lacking the last 100 amino acids failed to promote translocation at a detectable level (Fig. 4 B&D). These data indicate that the Dot/Icm transporter is capable of recognizing diverse features in the C-terminal portions of its substrates and that in some cases such differences do not affect translocation efficiencies of the proteins.

## Discussion

**Figure 5:**
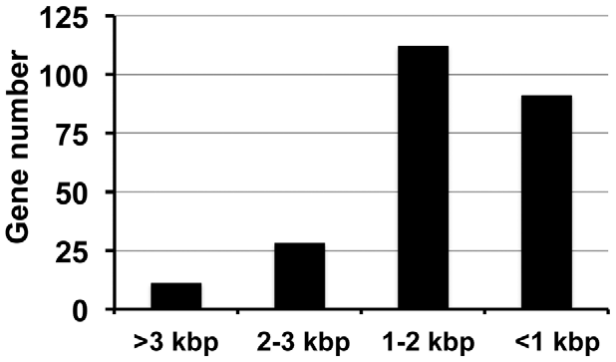
The distribution of all verified Dot/Icm substrates according to their length. Proteins experimentally shown as substrates of the Dot/Icm transporter were collected and sorted according to the length of the gene, which were then divided into four groups: I, genes larger than 3 kbp; II, genes ranging between 2 to 3 kbp; III, genes ranging between 1 to 2 kbp and IV, genes smaller than 1 kbp.

Analyses of the functions of proteins delivered into host cells by specialized transporters have provided great insights into the biology of both the pathogens and their hosts. Earlier studies that combined appropriate reporter systems and genetic screens and/or bioinformatics analyses have identified a large of number of protein substrates translocated by the *L. pneumophila* Dot/Icm system. Although effective, these strategies do not allow truly unbiased screens. In this study, we used a comprehensive method to test Dot/Icm-dependent translocation of hypothetical proteins larger than 100 amino acids in the genome of *L. pneumophila* strain Philadelphia 1. Our efforts had added 70 novel proteins to the Dot/Icm substrate inventory, which with previously proteins, have expanded experimentally confirmed Dot/Icm substrates to 275 (Table S4). Thus, judging by the number of proteins transferred, the *L. pneumophila* Dot/Icm system is arguably the most prolific bacterial translocator, whose substrates are more than five times of the effectors secreted by the Hrp type III secretion system of *Pseudomonas syringae*, a plant pathogen known to have a large repertoire of effectors [44]. Many Dot/Icm substrates identified by an earlier genetic method are larger than 100 kDa [10]. With a more complete list of its substrates, we analyzed the size distribution of these proteins. Surprisingly, only 39 genes are larger than 2 kbp of which 12 are more than 3 kbp (Fig. 5). The majority of the genes are shorter than 2 kbp, with 113 sizing between 1 to 2 kbp and 91 shorter than 1 kbp (Fig. 5). Thus, the length of most of the substrate genes is about 1 kbp, typical for proteins of bacterial origins [45]. That many substrates genes identified in an earlier genetic screen are longer than 2 kbps [10] may be a result of high probability of generating in-frame fusions of longer genes in the random library used for bacterial two-hybrid screenings.

Type IV transporter-mediated protein translocation is determined by at least three factors: Components of the transporter involved in recognizing the signals, signals encoded by amino acids embedded in the carboxyl portion of the substrates and chaperones responsible for proper folding of the substrate. In *L. pneumophila*, DotF, an important component of the Dot/Icm system interacts with a set of substrates [10], but whether this protein is involved in recognizing the substrates by interacting with patches of amino acids important for translocation is unknown. The roles played by the chaperones IcmS/IcmW in substrate translocation probably is by inducing conformational changes [46], which presumably will allow the translocation signals more properly exposed to the transporter. Accumulating evidence suggests that signals residing in the carboxyl end of Dot/Icm substrates are quite diverse. For examples, a hydrophobic residue located at -3 or -4 position is present in many Dot/Icm substrates and has been shown to be important for the translocation of RalF [15]. More recently, Huang et al showed that an E-block motif is important for the translocation of a subset of substrates [12]. Further, our results have revealed Lpg2844, a protein with a carboxyl amino acid composition highly different from most substrates can be translocated at high efficiencies (Fig. 4). Thus, it appears that in *L. pneumophila,* successful translocation does not need the presence of all known signals.

Residues important for protein translocation can contribute to substrate recognition by forming structural entities that directly bind to the transporter or by indirectly involved in this process by supporting the formation of such structures. Further experiments aiming at the interactions between transporter components and the substrates are required to assign specific roles to these amino acids in translocation. Clearly, the highly diverse translocation signals would allow a transporter to accommodate structurally different substrates, which is in great agreement with the large substrate pool possessed by the Dot/Icm system. Moreover, because the Dot/Icm system is functioning in actively replicating intracellular bacteria [47], translocation signals can determine the amount of a protein delivered into host cells, thus contributing to temporal control of effector activity during infection.

Although comprehensive, our direct screen method was limited by a number of factors and clearly was not exhaustive: First, we only examined proteins larger than 100 amino acids, yet at least one substrate, Lpg0045 (70 aa) is smaller than this threshold (Kubori et al., 2008). Second, our library may have excluded candidates annotated as proteins with detectable homology to proteins of known activity. Third, the β-lactamase fusion reporter may not work for some substrates. Fourth, some substrates may be translocated at efficiencies beyond the detection sensitivity of the β-lactamase assay. Finally, some of the β-lactamase fusions in our library may not be expressed at levels sufficient for detectable translocation. However, the number of false negative genes resulting from this reason, if any, should be low because high transfer efficiencies do not necessarily correlate with high protein levels (Fig. 3). Further, the β-lactamase fusions of 27 examined transfer-deficient genes all expressed at readily detectable levels (Fig. S1). In support of these potential limitations, 8 proteins capable of promoting the translocation of SidCΔC100 are not detectably positive in our assay (Table S5 and Ref. [12]). With the exception of lpg0926, the β-lactamase fusions of the other seven genes are stably expressed in *L. pneumophila* (data not shown). We further examined the translocation of this protein using the Cya fusion method; no translocation was detected despite the fusion was stably made in *L. pneumophila* (data not shown). The reasons for these discrepancies can be multiple. It is possible that some translocation signals still present in SidCΔC100, which would aid the transfer of proteins harboring low efficient transferring signals. Clearly, these potential limitations could be applied to each of the available reporter systems used to measure protein translocation. Nevertheless, that we have re-identified 94 of the substrates reported in the last several years indicates the reliability of the β-lactamase reporter system. Taken together, these observations suggest that the repertoire of the Dot/Icm substrate will likely continue to expand as more saturated screens are performed and/or more sensitive reporter systems are developed to detect translocation.

This extremely large substrate repertoire may explain the observation that deletion mutants lacking one or more of these genes rarely exhibit significant intracellular growth defect, probably due to functional redundancy exerted by subsets of these proteins [10]. Or, the accumulation of these effectors may be a result of the challenges faced by the bacterium to colonize phylogenetically diverse amoebae host in its natural niches. Alternatively, these proteins may not necessarily contribute to intracellular bacterial growth, the most common phenotype examined in the study of *L. pneumophila* pathogenesis. Instead, they may help the host adapt to challenges such as those brought by detrimental environmental changes and the fluctuations in nutrient supplies. Regardless of the reason, future studies directed to the elucidation of the biochemical and cell biological activities of these substrates will undoubtedly contribute greatly to our understanding the biology of both the pathogen and its hosts.

## Materials and Methods

### Bacterial strains, cell culture and media

The *L. pneumophila* strains used are derivatives of the strain Lp02 (*thyA Δ(hsdR-lvh) rpsL*) [48], and were grown on charcoal-yeast extract (CYE) solid medium or ACES-yeast extract (AYE) [48]; Lp03 contains the *dotA3* mutation [48]. The growth media were supplemented with thymidine at 100 µg/ml when appropriate. For infection experiments, the *L. pneumophila* strains used in all assays were grown to post-exponential phase (OD_600_≈3.4–3.7) unless stated otherwise. Antibiotics were used at the following concentrations with *E. coli* strains: ampicillin 100 µg/ml and chloramphenicol, 30 µg/ml. For *L. pneumophila* strains, chloramphenicol was used at 5 µg/ml. U937 cells were cultured in RPMI medium supplemented with 10% FBS prior to being induced by phorbol myristate acetate (PMA) (0.1 µg/ml). For assays in 96-well microtiter plates, differentiated U937 cells were plated in 96-well having optically clear bottoms in a density of 1×10^5^/well.

### Antibody and Western blotting

The antibody against β-lactamase was purchased from Abcam (Cambridge, MA). The antibody specific for the isocitrate dehydrogenase (ICDH) of *L. pneumophila* was described elsewhere [20]. To detect the expression of the fusion proteins in *L. pneumophila*, strains harboring the plasmids were grown in CYE medium supplemented with thymidine (200 mg/ml), chloramphenicol 5 mg/ml and IPTG (0.5 mM) to post exponential phase (OD_600_ = 3.4–3.7). Cells equal to 1 OD unit were withdrawn and dissovled in 200 ml of SDS loading buffer. After boiling for 5 min, cleared supernatant was resolved by SDS-PAGE. Separated proteins were transferred onto nitrocellulose membranes and proteins were detected by Western blot using an appropriate IRDye infrared secondary antibody (Li-Cor's Biosciences Lincoln, Nebraska, USA) and the signals were detected with an Odyssey infrared imaging system as described [20].

### Construction of β-lactamase fusion library

To accommodate the fusion of genes to the β-lactamase gene as *Bam*HI/*Sal*I DNA fragments in the first open reading frame, we inserted a DNA fragment obtained from annealing oligos 5′-CGGATCCCTGCAGGCGGCCGCGTCGACT-3′ and 5′-CATGGCCTAGGGACGTCCGCCGGCGCAGCTGAGATC-3′ into *Kpn*I and *Xba*I digested pDXC61[17] to give pDXC61M. To make the fusion library, open reading frames larger than 300 base pairs that code for hypothetical proteins were retrieved from the genome of *L. pneumophila* strain Philadelphia 1. 19-base primers were designed to amplify the entire gene by PCR with the Pfu UltraII high fidelity DNA polymerase (Agilent, Santa Clara, CA). In each case, DNA sequences recognized by the restriction enzymes *Bam*HI and *Sal*I were added to the 5′ and 3′ primers, respectively (Table S1). For genes whose sequences contain the *Bam*HI or *Sal*I recognition site, sequences for *Bgl*II or *Xho*I were added. For a number of genes that harbor one or more of these sites, other restriction enzymes were used (Table S1). After digestion with the appropriate restriction enzymes the PCR products were inserted into similarly digested pDXC61M. Plasmids containing correct inserts were introduced into *L. pneumophila* strains by electroporation. To make β-lactamase fusions with specific regions of genes, the target regions were amplified by PCR with the appropriate primers (Table S6) and were inserted into pDXC61M as described above.

### Screen for fusions that transfer the β-lactamase into mammalian cells

To test Dot/Icm-dependent transfer of the fusion proteins into host cells, *L. pneumophila* strains expressed the fusions grown to post exponential phase in the presence of 0.5 mM IPTG were used to infect monolayers of U937 cells seeded in 96-well plates at an MOI of 20. One hour after infection, the CCF4-AM substrates (Invitrogen, Carlsbad, CA) were mixed with medium in the wells. After further incubation for 2 hours at 25°C, infected cells were visually inspected under a Nikon IX-80 fluorescence microscope equipped with a β-lactamase FL-Cube (U-N41031, Chroma Technology Corp, Bellows Falls, VT). Images of infected cells were obtained by a DP-72 color fluorescence camera (Olympus). The percentage of infected cells was determined by counting the number of cells emiting blue fluorescence in specified areas of the wells. Experiments were performed in triplicate and in each sample and at least 300 cells were counted.

## Supporting Information

Figure S1 Expression of β-lactamase fusions in representative strains exhibiting undetectable protein translocation. *L. pneumophila* strains harboring fusions of β-lactamase to the indicated genes were grown in liquid medium containing 0.5 mM of IPTG to post-exponential phase (OD_600_ = 3.5–4.2). Cells corresponding to one OD unit were withdrawn and solubilized with 200 µl of SDS sample buffer. Ten µl of cleared supernatant were resolved in SDS-PAGE, proteins transferred onto nitrocellulose membranes were probed with indicated antibodies and detected with an Odyssey image system.
(PDF)

Table S1 Primers used for the construction of the β-lactamase fusion library.
(XLS)

Table S2 Characteristics of Dot/Icm substrates identified in this study.
(DOC)

Table S3 Experimentally confirmed substrates re-identified in this study.
(DOC)

Table S4 Experimentally confirmed protein substrates of the Dot/Icm transporter.
(DOC)

Table S5 Expression of proteins fusions for candidates positive for translocation in the SidCΔC100 assay but negative in the β-lactamase reporter assay.
(DOC)

Table S6 Primers for construction of deletion mutants of Lpg2844.
(DOC)
