# Supplementary figures and images for: Comprehensive Identification of Protein Substrates of the Dot/Icm Type IV Transporter of Legionella pneumophila
Source: PLoS One. 2011 Mar 9;6(3):e17638. doi: 10.1371/journal.pone.0017638 (PMC3052360; doi:10.1371/journal.pone.0017638)

Fig. S1

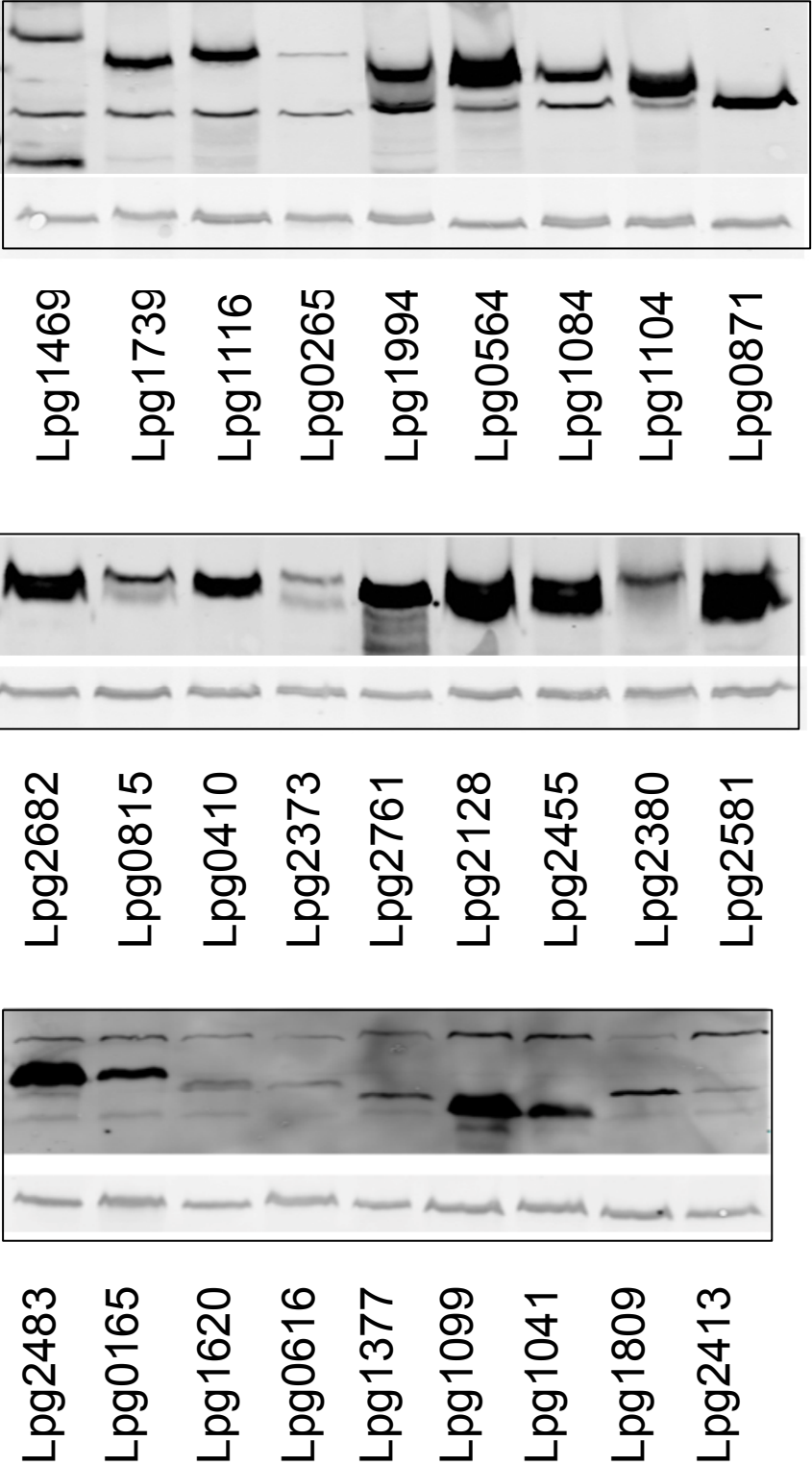

Supplement: Figure S1 — Expression of β-lactamase fusions in representative strains exhibiting undetectable protein translocation. L. pneumophila strains harboring fusions of β-lactamase to the indicated genes were grown in liquid medium containing 0.5 mM of IPTG to post-exponential phase (OD600 = 3.5–4.2). Cells corresponding to one OD unit were withdrawn and solubilized with 200 µl of SDS sample buffer. Ten µl of cleared supernatant were resolved in SDS-PAGE, proteins transferred onto nitrocellulose membranes were probed with indicated antibodies and detected with an Odyssey image system. (PDF) [file pone.0017638.s001.pdf]
