# Supplementary material for: Comprehensive Identification of Protein Substrates of the Dot/Icm Type IV Transporter of Legionella pneumophila
Source: PLoS One. 2011 Mar 9;6(3):e17638. doi: 10.1371/journal.pone.0017638 (PMC3052360; doi:10.1371/journal.pone.0017638)
Supplement: Table S2 — Characteristics of Dot/Icm substrates identified in this study. (DOC) [file pone.0017638.s003.doc]

Table S2 Characteristics of Dot/Icm substrates identified in this study

|  |  | Lpg number | Size（base pair） | Translocation Efficiency | Putative motif/domain | Most Proximate effector | Paralog in *L. pneumophila* Philadelphia-1 | Homolog in other *L. pneumophila* strains | | |
| --- | --- | --- | --- | --- | --- | --- | --- | --- | --- | --- |
|  |  |  |  |  |  |  |  | Paris | Lens | Corby |
|  | 1 | Lpg0021 | 480 | 5% | N/A | N/A | N/A | Lpp0021 | Lpl0022 | LPC_0022 |
| 2 | 2 | Lpg0046 | 399 | 5% | N/A | Lpg0045 | N/A | Lpp0047 | Lpl0045 | LPC_0048 |
| 4 | 3 | Lpg0130 | 2496 | 80% | N/A | N/A | N/A | Lpp0145 | Lpl0130 | LPC_0151 |
| 5 | 4 | Lpg0172 | 726 | 10% | N/A | Lpg0171 | N/A | Lpp0234 | N/A | LPC_0253 |
| 6 | 5 | Lpg0181 | 912 | 50% | N/A | N/A | N/A | Lpp0245 | Lpl0244 | LPC_0265 |
| 9 | 6 | Lpg0260 | 399 | 5-10% | N/A | N/A | N/A | Lpp0332 | Lpl0313 | LPC_0337 |
| 10 | 7 | Lpg0364 | 306 | 5% | N/A | Lpg0365 | N/A | Lpp0429 | Lpl0425 | N/A |
| 11 | 8 | Lpg0375 | 600 | 5-10% | N/A | Lpg0376 | N/A | Lpp0442 | Lpl0418 | LPC-2968 |
| 12 | 9 | Lpg0405 | 591 | 5-10% | N/A | N/A | N/A | Lpp0471 | Lpl0447 | LPC-2939 |
|  | 10 | Lpg0716 | 1014 | 50% | N/A | N/A | N/A | Lpp0872 | Lpl0753 | LPC_2577 |
| 13 | 11 | Lpg0796 | 651 | 50% | N/A | N/A | N/A | Lpp0859 | Lpl1823 | N/A |
| 14 | 12 | Lpg0967 | 594 | 50-60% | N/A | SidK(Lpg0968) | N/A | Lpp1029 | N/A | LPC_2320 |
| 15 | 13 | Lpg1083 | 684 | 30% | N/A | N/A | N/A | N/A | N/A | N/A |
| 17 | 14 | Lpg1106 | 1275 | 50% | N/A | N/A | N/A | Lpp1105 | Lpl1105 | LPC_2149 |
| 18 | 15 | Lpg1124 | 591 | 5-10% | N/A | N/A | N/A | Lpp1125 | Lpl1129 | LPC_0582 |
| 19 | 16 | Lpg1137 | 969 | 10-20% | Translocase of the mitochondrial inner membrane  (TIM) | N/A | N/A | Lpp1139 | Lpl1144 | LPC_0601 |
| 20 | 17 | Lpg1147 | 504 | 50% | N-acetyltransferase super family | Lpg1148 | Lpg2420 | Lpp2313 | Lpl1153 | LPC_0610 |
| 21 | 18 | Lpg1171 | 420 | 80% | N/A | N/A | N/A | Lpp1173 | Lpl1179 | LPC_0673 |
| 22 | 19 | Lpg1449 | 2613 | 50% | DUS-FNM phosphate-binding super family^1^ | N/A | N/A | Lpp1404 | N/A | N/A |
| 24 | 20 | Lpg1453 | 519 | 5-10% | N/A | N/A | N/A | Lpp1409 | Lpl1591 | N/A |
|  | 21 | Lpg1484 | 810 | 20-50% | N/A | Lpg1483 (LegK1) | N/A | Lpp1440 | Lpl1544 | LPC_0899 |
| 25 | 22 | Lpg1578 | 450 | 5-10% | N/A | N/A | N/A | N/A | N/A | LPC_1002 |
| 27 | 23 | Lpg1639 | 1317 | 20% | N/A | N/A | N/A | Lpp1609 | Lpl1387 | N/A |
| 28 | 24 | Lpg1654 | 1119 | 40% | Tetratricopeptide repeat | N/A | N/A | Lpp1625 | N/A | LPC_1084 |
| 29 | 25 | Lpg1661 | 1119 | 5-10% | Acyltransferase family | Lpg1660 | N/A | Lpp1632 | Lpl1626 | LPC_1091 |
|  | 26 | Lpg1666 | 1404 | 50% | N/A | N/A | Lpg1667 | Lpp1637 | Lpl1631 | LPC_1096 |
|  | 27 | Lpg1667 | 1392 | 80% | IgA Peptidase M64^2^ | N/A | Lpg1666 | Lpp1638 | Lpl1632 | LPC_1097 |
| 30 | 28 | Lpg1670 | 891 | 5-10% | N/A | N/A | N/A | Lpp1642 | Lpl1635 | N/A |
|  | 29 | Lpg1684 | 1398 | 80% | D123 super family^3^ | Lpg1683 | N/A | N/A | N/A | N/A |
|  | 30 | Lpg1685 | 870 | 40% | N/A | N/A | N/A | N/A | N/A | LPC_1116 |
| 31 | 31 | Lpg1692 | 1311 | 80% | N/A | N/A | N/A | Lpp1330 | N/A | N/A |
|  | 32 | Lpg1716 | 459 | 20% | N/A | Lpg1717 | N/A | N/A | Lpl1675 | LPC_1146 |
| 32 | 33 | Lpg1776 | 648 | 5-10% | N/A | N/A | N/A | Lpp1740 | Lpl1740 | N/A |
| 33 | 34 | Lpg1803 | 936 | 20% | Rho_GEF^4^ | N/A | N/A | Lpp1766 | Lpl1766 | N/A |
| 36 | 35 | Lpg1888 | 1332 | 5% | PLDc Super family^5^ | N/A | N/A | Lpp1885 | Lpl1850 | LPC_1336 |
| 37 | 36 | Lpg1907 | 1806 | 90% | Lipase Family | N/A | N/A | Lpp1882 | Lpl1871 | LPC_1361 |
| 49 | 37 | Lpg1924 | 2793 | 80% | N/A | N/A | N/A | Lpp1899 | Lpl1888 | LPC_1378 |
| 39 | 38 | Lpg1959 | 1995 | 70% | N/A | Lpg1958 | N/A | Lpp1941 | Lpl1932 | LPC_1436 |
| 40 | 39 | Lpg1986 | 2853 | 90% | TraI_TIGR  Domain^6^ | N/A | N/A | Lpp1967 | Lpl1961 | LPC_1469 |
| 41 | 40 | Lpg2050 | 1059 | 10-20% | PKC_like family, CRIK subfamily^7^ | N/A | N/A | Lpp2033 | Lpl2028 | LPC_1536 |
| 42 | 41 | Lpg2148 | 1281 | 90% | N/A | Lpg2149 | Lpg2147 | Lpp2087 | Lpl2076 | LPC_1597 |
| 43 | 42 | Lpg2149 | 360 | 20-30% | N/A | Lpg2148 | N/A | Lpp2088 | Lpl2077 | LPC_1598 |
| 44 | 43 | Lpg2223 | 1224 | 80% | N/A | Lpg2222 | N/A | Lpp2175 | Lpl2149 | LPC_1691 |
| 46 | 44 | Lpg2239 | 3858 | 80% | N/A | N/A | N/A | Lpp2192 | N/A | N/A |
| 47 | 45 | Lpg2271 | 651 | 80% | N/A | N/A | N/A | Lpp2225 | N/A | LPC_1740 |
| 59 | 46 | Lpg2359 | 444 | 5-10% | N/A | N/A | N/A | Lpp2308 | Lpl2281 | LPC_1828 |
| 50 | 47 | Lpg2370 | 939 | 20% | HipA Super family^8^ | N/A | Lpg2380 | N/A | Lpl2292 | LPC_2112 |
| 51 | 48 | Lpg2372 | 1269 | 5% | N/A | N/A | N/A | Lpp3009 | N/A | LPC_3248 |
| 52 | 49 | Lpg2382 | 1455 | 15% | N/A | N/A | N/A | Lpp2444 | Lpl2300 | LPC_2108 |
| 53 | 50 | Lpg2434 | 492 | 5% | Cupin super family | Lpg2433 | N/A | Lpp2501 | Lpl2355 | LPC_2042 |
| 58 | 51 | Lpg2443 | 558 | 5-10% | N/A | N/A | N/A | Lpp2510 | Lpl2363 | LPC_2033 |
| 59 | 52 | Lpg2461 | 639 | 5-10% | N/A | N/A | N/A | Lpp2527 | Lpl2380 | LPC_2015 |
| 60 | 53 | Lpg2505 | 888 | 20-30% | N/A | N/A | N/A | Lpp2573 | Lpl2427 | LPC_1966 |
|  | 54 | Lpg2508 | 2424 | 10% | N/A | N/A | Lpg2155 | Lpp2576 | Lpl2430 | LPC_1604 |
| 61 | 55 | Lpg2538 | 1416 | 75% | N/A | Lpg2539 | N/A | Lpp2604 | Lpl2459 | LPC_1930 |
| 63 | 56 | Lpg2539 | 408 | 20% | N/A | Lpg2538 | N/A | Lpp2605 | Lpl2460 | LPC_1929 |
| 64 | 57 | Lpg2546 | 1410 | 10-20% | N/A | N/A | N/A | Lpp2615 | N/A | LPC_1919 |
|  | 58 | Lpg2555 | 855 | 30% | HAD-like superfamily^9^ | Lpg2556 | N/A | Lpp2625 | Lpl2480 | LPC_1908 |
| 65 | 59 | Lpg2628 | 753 | 50% | N/A | N/A | N/A | Lpp2681 | Lpl2553 | LPC_0513 |
| 66 | 60 | Lpg2637 | 1212 | 75% | N/A | N/A | N/A | Lpp2690 | Lpl2562 | LPC_0503 |
| 67 | 61 | Lpg2692 | 531 | 5-10% | N/A | N/A | N/A | Lpp2746 | Lpl2619 | LPC_0444 |
| 69 | 62 | Lpg2745 | 1968 | 60% | N/A | Lpg2744 | N/A | Lpp2801 | Lpl2670 | LPC_0385 |
| 70 | 63 | Lpg2826 | 1734 | 90% | Peptidase C26 | N/A | N/A | N/A | Lpl2741 | LPC_3113 |
| 72 | 64 | Lpg2832 | 1641 | 40% | N/A | Lpg2831 | N/A | Lpp2889 | Lpl2744 | LPC_3116 |
| 73 | 65 | Lpg2844 | 1086 | 90% | N/A | N/A | N/A | Lpp2903 | Lpl2756 | LPC_3128 |
| 74 | 66 | Lpg2885 | 555 | 20% | N/A | N/A | N/A | Lpp2944 | Lpl2798 | LPC_3171 |
| 75 | 67 | Lpg2888 | 1914 | 10-20% | N/A | N/A | N/A | Lpp2947 | Lpl2801 | LPC_3174 |
| 76 | 68 | Lpg2912 | 1488 | 80% | N/A | N/A | N/A | Lpp2980 | Lpl2830 | LPC_3214 |
|  |  |  |  |  |  |  |  |  |  |  |
| 77 | 69 | Lpg2936 | 735 | 5-10% | RNA methyltransferase super family | N/A | N/A | Lpp3004 | Lpl2865 | LPC_3243 |
| 80 | 70 | Lpg3000 | 1839 | 50% | N/A | Lpg2999 | N/A | Lpp3072 | Lpl2728 | LPC_3316 |

Notes:

| 97 |
| --- |

^1^ DUS-FNM phosphate-binding super family: Dihydrouridine synthase-like (DUS-like) FMN-binding domain

^2^ IgA Peptidase M64: Highly selective metallo-endopeptidases that cleaves IgA

^3^ D123 superfamily: This family contains a number of eukaryotic D123 proteins

^4^ Rho GEF: Rho GTPase Guanine exchange factor

^5^ PLDc family: Phospholipase D family

^6^ TraI TIGR Domain: Conjugative transfer relaxase protein TraI

^7^ PKC like super family: Protein Kinase C superfamily

^8^ HipA family: High-frequency-persistence mutants (Hip) A family

^9^ HAD like superfamily: Haloacid dehalogenase-like hydrolases superfamily

^10^ ZnMc superfamily: Zinc-dependent metalloprotease, astacin like subfamily or peptidase family M12A
