# Supplementary material for: Comprehensive Identification of Protein Substrates of the Dot/Icm Type IV Transporter of Legionella pneumophila
Source: PLoS One. 2011 Mar 9;6(3):e17638. doi: 10.1371/journal.pone.0017638 (PMC3052360; doi:10.1371/journal.pone.0017638)
Supplement: Table S3 — Experimentally confirmed substrates re-identified in this study. (DOC) [file pone.0017638.s004.doc]

Table S3 Experimentally confirmed substrates re-identified in this study

| Number | Alias | Lpg number | length (bp) | Reference |
| --- | --- | --- | --- | --- |
| 1 | ravA | Lpg0008 | 1260 | [1] |
| 2 | ravB | Lpg0030 | 915 | [1] |
| 3 | ceg2 | Lpg0059 | 1107 | [2] |
| 4 | N/A | Lpg0081 | 1326 | [3] |
| 5 | ceg4 | Lpg0096 | 1194 | [2] |
| 6 | N/A | Lpg0160 | 978 | [1] |
| 7 | ceg5 | Lpg0191 | 954 | [2] |
| 8 | N/A | Lpg0195 | 1005 | [1] |
| 9 | N/A | Lpg0196 | 1275 | [1] |
| 10 | ravG | Lpg0210 | 624 | [1] |
| 11 | ceg7 | Lpg0227 | 1107 | [4] |
| 12 | ceg9 | Lpg0246 | 726 | [5] |
| 13 | ceg10 | Lpg0294 | 693 | [3] |
| 14 | ceg11 | Lpg0401 | 675 | [1] |
| 15 | ceg15 | Lpg0439 | 1050 | [1] |
| 16 | lem3 | Lpg0696 | 1713 | [2] |
| 17 | ravH | Lpg0733 | 1499 | [1] |
| 18 | ceg18 | Lpg0898 | 732 | [4] |
| 19 | ravJ | Lpg0944 | 1176 | [1] |
| 20 | N/A | Lpg0963 | 1242 | [1] |
| 21 | sidK | Lpg0968 | 1722 | [6] |
| 22 | ravK | Lpg0969 | 741 | [1] |
| 23 | ravM | Lpg1109 | 2061 | [1] |
| 24 | lem6 | Lpg1120 | 1767 | [2] |
| 25 | Ceg19 | Lpg1121 | 771 | [2] |
| 26 | ravO | Lpg1129 | 1560 | [1] |
| 27 | lem7 | Lpg1145 | 2310 | [2] |
| 28 | N/A | Lpg1148 | 1512 | [3] |
| 29 | ravQ | Lpg1154 | 1083 | [1] |
| 30 | ravR | Lpg1166 | 2013 | [1] |
| 31 | ravS | Lpg1183 | 1893 | [1] |
| 32 | N/A | Lpg1273 | 1065 | [3] |
| 33 | lem8 | Lpg1290 | 1587 | [2] |
| 34 | ravT | Lpg1316 | 1023 | [1] |
| 35 | ravW | Lpg1317 | 888 | [1] |
| 36 | vpdC | Lpg1426 | 2665 | [7] |
| 37 | ravX | Lpg1489 | 1032 | [1] |
| 38 | lem9 | Lpg1491 | 1242 | [2] |
| 39 | lem10 | Lpg1496 | 1797 | [2] |
| 40 | ravY | Lpg1551 | 747 | [1] |
| 41 | legC6 | Lpg1588 | 2019 | [3] |
| 42 | lem11 | Lpg1598 | 1062 | [2] |
| 43 | lem12 | Lpg1625 | 393 | [2] |
| 44 | mavA | Lpg1687 | 1203 | [1] |
| 45 | N/A | Lpg1689 | 627 | [3] |
| 46 | N/A | Lpg1717 | 1689 | [3] |
| 47 | N/A | Lpg1751 | 1311 | [3] |
| 48 | mavB | Lpg1752 | 648 | [1] |
| 49 | rvfA | Lpg1797 | 1281 | [1] |
| 50 | mavU | Lpg1798 | 1197 | [1] |
| 51 | lem14 | Lpg1851 | 663 | [2] |
| 52 | legLC8 | Lpg1890 | 1722 | [3] |
| 53 | lem17 | Lpg1949 | 1341 | [2] |
| 54 | legC4 | Lpg1953 | 2283 | [8] |
| 55 | legL5 | Lpg1958 | 1629 | [3] |
| 56 | lirC | Lpg1963 | 2100 | [9] |
| 57 | lirD | Lpg1964 | 1311 | [9] |
| 58 | lirE | Lpg1965 | 2967 | [9] |
| 59 | lirF | Lpg1966 | 1566 | [9] |
| 60 | lem18 | Lpg1969 | 1908 | [10] |
| 61 | lem19 | Lpg2166 | 1260 | [2] |
| 62 | mavD | Lpg2199 | 633 | [1] |
| 63 | lem21 | Lpg2248 | 2235 | [2] |
| 64 | mavF | Lpg2351 | 921 | [1] |
| 65 | legL7 | Lpg2400 | 1065 | [8] |
| 66 | lem23 | Lpg2406 | 1098 | [2] |
| 67 | N/A | Lpg2407 | 306 | [3] |
| 68 | vpdA | Lpg2410 | 1998 | [7] |
| 69 | N/A | Lpg2420 | 552 | [1] |
| 70 | lem25 | Lpg2422 | 2622 | [2] |
| 71 | mavG | Lpg2424 | 1347 | [1] |
| 72 | mavH | Lpg2425 | 807 | [1] |
| 73 | ceg30 | Lpg2433 | 1761 | [2] |
| 74 | legA15 | Lpg2456 | 1416 | [8] |
| 75 | sidM | Lpg2464 | 1944 | [11] |
| 76 | mavJ | Lpg2498 | 933 | [1] |
| 77 | lem26 | Lpg2523 | 2340 | [2] |
| 78 | mavL | Lpg2526 | 1368 | [1] |
| 79 | N/A | Lpg2527 | 1677 | [3] |
| 80 | lem27 | Lpg2529 | 1719 | [2] |
| 81 | N/A | Lpg2541 | 834 | [12] |
| 82 | N/A | Lpg2552 | 1668 | [1] |
| 83 | mavM | Lpg2577 | 759 | [1] |
| 84 | Lem28 | Lpg2603 | 1305 | [2] |
| 85 | mavV | Lpg2638 | 1383 | [1] |
| 86 | wipA | Lpg2718 | 1563 | [13] |
| 87 | N/A | Lpg2744 | 1170 | [3] |
| 88 | lem29 | Lpg2804 | 1407 | [2] |
| 89 | mavN | Lpg2815 | 2052 | [1] |
| 90 | ceg34 | Lpg2826 | 1734 | [2] |
| 91 | legC8 | Lpg2862 | 1911 | [8] |
| 92 | N/A | Lpg2874 | 885 | [1] |
| 93 | mavO | Lpg2879 | 1752 | [1] |
| 94 | mavQ | Lpg2975 | 2616 | [1] |

N/A, not available.

Reference:

1. Huang L, Boyd D, Amyot WM, Hempstead AD, Luo ZQ, et al. (2010) The E Block motif is associated with *Legionella pneumophila* translocated substrates. Cell Microbiol 13: 227-245.

2. Burstein D, Zusman T, Degtyar E, Viner R, Segal G, et al. (2009) Genome-scale identification of *Legionella pneumophila* effectors using a machine learning approach. PLoS Pathog 5: e1000508.

3. Kubori T, Hyakutake A, Nagai H (2008) Legionella translocates an E3 ubiquitin ligase that has multiple U-boxes with distinct functions. Mol Microbiol 67: 1307-1319.

4. Altman E, Segal G (2008) The response regulator CpxR directly regulates expression of several *Legionella pneumophila* icm/dot components as well as new translocated substrates. J Bacteriol 190: 1985-1996.

5. Heidtman M, Chen EJ, Moy MY, Isberg RR (2008) Large-scale identification of *Legionella pneumophila* Dot/Icm substrates that modulate host cell vesicle trafficking pathways. Cell Microbiol.

6. Xu L, Shen X, Bryan A, Banga S, Swanson MS, et al. (2010) Inhibition of host vacuolar H+-ATPase activity by a *Legionella pneumophila* effector. PLoS Pathog 6: e1000822.

7. VanRheenen SM, Luo ZQ, O'Connor T, Isberg RR (2006) Members of a *Legionella pneumophila* family of proteins with ExoU (phospholipase A) active sites are translocated to target cells. Infect Immun 74: 3597-3606.

8. de Felipe KS, Pampou S, Jovanovic OS, Pericone CD, Ye SF, et al. (2005) Evidence for acquisition of Legionella type IV secretion substrates via interdomain horizontal gene transfer. J Bacteriol 187: 7716-7726.

9. Zusman T, Degtyar E, Segal G (2008) Identification of a hypervariable region containing new *Legionella pneumophila* Icm/Dot translocated substrates by using the conserved icmQ regulatory signature. Infect Immun 76: 4581-4591.

10. Ninio S, Celli J, Roy CR (2009) A *Legionella pneumophila* effector protein encoded in a region of genomic plasticity binds to Dot/Icm-modified vacuoles. PLoS Pathog 5: e1000278.

11. Machner MP, Isberg RR (2006) Targeting of host Rab GTPase function by the intravacuolar pathogen *Legionella pneumophila.* Dev Cell 11: 47-56.

12. Ivanov SS, Charron G, Hang HC, Roy CR (2010) Lipidation by the host prenyltransferase machinery facilitates membrane localization of *Legionella pneumophila* effector proteins. J Biol Chem 285: 34686-34698.

13. Ninio S, Zuckman-Cholon DM, Cambronne ED, Roy CR (2005) The Legionella IcmS-IcmW protein complex is important for Dot/Icm-mediated protein translocation. Mol Microbiol 55: 912-926.
