# Supplementary material for: Comprehensive Identification of Protein Substrates of the Dot/Icm Type IV Transporter of Legionella pneumophila
Source: PLoS One. 2011 Mar 9;6(3):e17638. doi: 10.1371/journal.pone.0017638 (PMC3052360; doi:10.1371/journal.pone.0017638)
Supplement: Table S4 — Experimentally confirmed protein substrates of the Dot/Icm transporter. (DOC) [file pone.0017638.s005.doc]

Table S4 Experimentally confirmed protein substrates of the Dot/Icm transporter

|  | Lpg number | Alias | Size | Reference |
| --- | --- | --- | --- | --- |
| 1 | Lpg0008 | ravA | 1260 | [1] |
| 2 | Lpg0012 | CegC1 | 1575 | [2] |
| 3 | Lpg0021 | N/A | 480 | This study |
| 4 | Lpg0030 | ravB | 915 | [1] |
| 5 | Lpg0038 | LegA10 | 1521 | [3] |
| 6 | lpg0041 | N/A | 2187 | [2] |
| 7 | Lpg0045 | N/A | 210 | [4] |
| 8 | Lpg0046 | N/A | 399 | This study |
| 9 | Lpg0059 | Ceg2 | 1107 | [5] |
| 10 | Lpg0080 | Ceg3 | 765 | [5] |
| 11 | Lpg0081 | N/A | 1326 | [4] |
| 12 | Lpg0090 | Lem1 | 4125 | [5] |
| 13 | Lpg0096 | Ceg4 | 1194 | [5] |
| 14 | Lpg0103 | VipF | 858 | [6] |
| 15 | Lpg0126 | CegC2 | 3306 | [2] |
| 16 | Lpg0130 | N/A | 2496 | This study |
| 17 | Lpg0160 | ravD | 978 | [1] |
| 18 | Lpg0170 | ravC | 807 | [1] |
| 19 | Lpg0171 | LegU1 | 564 | [3] |
| 20 | Lpg0172 | N/A | 726 | This study |
| 21 | Lpg0181 | N/A | 912 | This study |
| 22 | Lpg0191 | Ceg5 | 954 | [5] |
| 23 | Lpg0195 | ravE | 1005 | [1] |
| 24 | Lpg0196 | ravF | 1275 | [1] |
| 25 | Lpg0210 | ravG | 624 | [1] |
| 26 | Lpg0227 | Ceg7 | 1104 | [2] |
| 27 | Lpg0234 | SidE | 4542 | [7] |
| 28 | Lpg0240 | Ceg8 | 777 | [5] |
| 29 | Lpg0246 | Ceg9 | 723 | [8] |
| 30 | Lpg0260 | N/A | 399 | This study |
| 31 | Lpg0276 | LegG2 | 1497 | [3] |
| 32 | Lpg0284 | Ceg10 | 1122 | [9] |
| 33 | Lpg0285 | Lem2 | 684 | [5] |
| 34 | Lpg0294 | N/A | 693 | [5] |
| 35 | Lpg0364 | N/A | 306 | This study |
| 36 | Lpg0365 | N/A | 2688 | [4] |
| 37 | Lpg0375 | N/A | 600 | This study |
| 38 | Lpg0376 | SdhA | 4287 | [7] |
| 39 | Lpg0390 | VipA | 1056 | [6] |
| 40 | Lpg0401 | Ceg11 | 675 | [1] |
| 41 | Lpg0402 | LegA9 | 1701 | [3] |
| 42 | Lpg0403 | LegA7 | 672 | [10] |
| 43 | Lpg0405 | N/A | 591 | This study |
| 44 | Lpg0422 | LegY | 1347 | [3] |
| 45 | Lpg0436 | LegA11 | 807 | [3] |
| 46 | Lpg0437 | Ceg14 | 2001 | [5] |
| 47 | Lpg0439 | Ceg15 | 1050 | [1] |
| 48 | Lpg0483 | LegA12 | 1485 | [3] |
| 49 | Lpg0515 | LegD2 | 930 | [3] |
| 50 | Lpg0518 | N/A | 846 | [4] |
| 51 | Lpg0519 | Ceg17 | 2214 | [5] |
| 52 | Lpg0621 | SidA | 1422 | [7] |
| 53 | Lpg0634 | N/A | 1347 | [4] |
| 54 | Lpg0642 | WipB | 1572 | [11] |
| 55 | Lpg0695 | LegA8 | 2847 | [3] |
| 56 | Lpg0696 | Lem3 | 1713 | [5] |
| 57 | Lpg0716 | N/A | 1014 | This study |
| 58 | Lpg0733 | ravH | 1449 | [1] |
| 59 | Lpg0796 | N/A | 651 | This study |
| 60 | Lpg0898 | Ceg18 | 729 | [2] |
| 61 | Lpg0926 | ravI | 1011 | [1] |
| 62 | Lpg0940 | LidA | 2190 | [12] |
| 63 | Lpg0944 | ravJ | 1176 | [1] |
| 64 | Lpg0945 | LegL1 | 888 | [3] |
| 65 | Lpg0963 | N/A | 1242 | [4] |
| 66 | Lpg0967 | N/A | 594 | This study |
| 67 | Lpg0968 | SidK | 1722 | [13] |
| 68 | Lpg0969 | ravK | 741 | [1] |
| 69 | Lpg1083 | N/A | 684 | This study |
| 70 | Lpg1101 | Lem4 | 969 | [5] |
| 71 | Lpg1106 | N/A | 1275 | This study |
| 72 | Lpg1108 | ravL | 882 | [1] |
| 73 | Lpg1109 | ravM | 2061 | [1] |
| 74 | Lpg1110 | Lem5 | 696 | [5] |
| 75 | Lpg1111 | ravN | 639 | [1] |
| 76 | Lpg1120 | Lem6 | 1767 | [5] |
| 77 | Lpg1121 | Ceg19 | 771 | [5] |
| 78 | Lpg1124 | N/A | 591 | This study |
| 79 | Lpg1129 | ravO | 1560 | [1] |
| 80 | Lpg1137 | N/A | 969 | This study |
| 81 | Lpg1144 | CegC3 | 504 | [2] |
| 82 | Lpg1145 | Lem7 | 2310 | [5] |
| 83 | Lpg1147 | N/A | 504 | This study |
| 84 | Lpg1148 | N/A | 1512 | [4] |
| 85 | Lpg1152 | ravP | 857 | [1] |
| 86 | Lpg1154 | ravQ | 1083 | [1] |
| 87 | Lpg1158 | N/A | 768 | [4] |
| 88 | Lpg1166 | ravR | 2013 | [1] |
| 89 | Lpg1171 | N/A | 420 | This study |
| 90 | Lpg1183 | ravS | 1893 | [1] |
| 91 | Lpg1227 | VpdB | 1794 | [14] |
| 92 | Lpg1273 | N/A | 1065 | [4] |
| 93 | Lpg1290 | Lem8 | 1587 | [5] |
| 94 | lpg1312 | legC1 | 3405 | [3] |
| 95 | Lpg1316 | ravT | 1023 | [1] |
| 96 | Lpg1317 | ravW | 888 | [1] |
| 97 | Lpg1328 | LegT | 2463 | [3] |
| 98 | Lpg1355 | SidG | 2922 | [7] |
| 99 | Lpg1426 | VpdC | 884 | [14] |
| 100 | Lpg1449 | N/A | 2613 | This study |
| 101 | Lpg1453 | N/A | 519 | This study |
| 102 | lpg1483 | LegK1 | 1587 | [3] |
| 103 | Lpg1484 | N/A | 810 | This study |
| 104 | Lpg1488 | LegC5 | 2595 | [3] |
| 105 | Lpg1489 | ravX | 1032 | [1] |
| 106 | Lpg1491 | Lem9 | 1242 | [5] |
| 107 | Lpg1496 | Lem10 | 1797 | [5] |
| 108 | Lpg1551 | ravY | 747 | [1] |
| 109 | Lpg1578 | N/A | 450 | This study |
| 110 | Lpg1588 | LegC6 | 2019 | [3] |
| 111 | Lpg1598 | Lem11 | 1062 | [5] |
| 112 | Lpg1602 | LegL2 | 1284 | [3] |
| 113 | Lpg1621 | Ceg23 | 1317 | [9] |
| 114 | Lpg1625 | Lem12 | 393 | [5] |
| 115 | Lpg1639 | N/A | 1317 | This study |
| 116 | Lpg1642 | SidB | 1251 | [7] |
| 117 | Lpg1654 | N/A | 1119 | This study |
| 118 | Lpg1660 | LegL3 | 1479 | [3] |
| 119 | Lpg1661 | N/A | 1119 | This study |
| 120 | Lpg1666 | N/A | 1404 | This study |
| 121 | Lpg1667 | N/A | 1392 | This study |
| 122 | Lpg1670 | N/A | 891 | This study |
| 123 | Lpg1683 | ravZ | 1509 | [1] |
| 124 | Lpg1684 | N/A | 1398 | This study |
| 125 | Lpg1685 | N/A | 870 | This study |
| 126 | Lpg1687 | mavA | 1203 | [1] |
| 127 | Lpg1689 | N/A | 624 | [4] |
| 128 | Lpg1692 | N/A | 1311 | This study |
| 129 | Lpg1701 | LegC3 | 1680 | [3] |
| 130 | Lpg1702 | PpeB | 1638 | [15] |
| 131 | Lpg1716 | N/A | 459 | This study |
| 132 | Lpg1717 | N/A | 1689 | [4] |
| 133 | Lpg1718 | LegAS4 | 1635 | [3] |
| 134 | Lpg1751 | N/A | 1311 | [4] |
| 135 | Lpg1752 | N/A | 648 | [1] |
| 136 | Lpg1776 | N/A | 648 | This study |
| 137 | Lpg1797 | rvfA | 1281 | [1] |
| 138 | Lpg1798 | marB | 1197 | [1] |
| 139 | Lpg1803 | N/A | 936 | This study |
| 140 | Lpg1851 | Lem14 | 663 | [5] |
| 141 | Lpg1884 | YlfB | 1215 | [16] |
| 142 | Lpg1888 | N/A | 1332 | This study |
| 143 | Lpg1890 | LegLC8 | 1722 | [3] |
| 144 | Lpg1907 | N/A | 1806 | This study |
| 145 | Lpg1924 | N/A | 2793 | This study |
| 146 | Lpg1933 | Lem15 | 615 | [5] |
| 147 | Lpg1947 | Lem16 | 750 | [5] |
| 148 | Lpg1948 | LegLC4 | 1005 | [3] |
| 149 | Lpg1949 | Lem17 | 1341 | [5] |
| 150 | Lpg1950 | RalF | 1122 | [17] |
| 151 | Lpg1953 | LegC4 | 2283 | [3] |
| 152 | Lpg1958 | LegL5 | 1629 | [3] |
| 153 | Lpg1959 | N/A | 1995 | This study |
| 154 | Lpg1960 | LirA | 771 | [18] |
| 155 | Lpg1962 | LirB | 564 | [18] |
| 156 | Lpg1963 | LirC | 2100 | [18] |
| 157 | Lpg1964 | LirD | 1311 | [18] |
| 158 | Lpg1965 | LirE | 2967 | [18] |
| 159 | Lpg1966 | LirF | 1566 | [18] |
| 160 | Lpg1969 | PieE | 1908 | [15] |
| 161 | Lpg1972 | PieF | 375 | [15] |
| 162 | Lpg1976 | LegG1 | 858 | [3] |
| 163 | Lpg1978 | SetA | 1932 | [8] |
| 164 | Lpg1986 | N/A | 2853 | This study |
| 165 | Lpg2050 | N/A | 1059 | This study |
| 166 | lpg2131 | LegA6 | 540 | [3] |
| 167 | Lpg2137 | LegK2 | 1614 | [3] |
| 168 | Lpg2144 | LegAU13 | 516 | [3] |
| 169 | Lpg2147 | mavC | 1449 | [1] |
| 170 | Lpg2148 | N/A | 1281 | This study |
| 171 | Lpg2149 | N/A | 360 | This study |
| 172 | Lpg2153 | SdeC | 4599 | [7] |
| 173 | Lpg2155 | SidJ | 2619 | [19] |
| 174 | lpg2156 | SdeB | 5178 | [18] |
| 175 | Lpg2157 | SdeA | 4518 | [7] |
| 176 | Lpg2166 | Lem19 | 1260 | [5] |
| 177 | Lpg2176 | LegS2 | 1824 | [3] |
| 178 | Lpg2199 | mavD | 633 | [1] |
| 179 | Lpg2200 | CegC4 | 534 | [2] |
| 180 | Lpg2215 | LegA2 | 1593 | [3] |
| 181 | Lpg2216 | Lem20 | 1770 | [5] |
| 182 | Lpg2223 | N/A | 1224 | This study |
| 183 | Lpg2224 | PpgA | 1779 | [15] |
| 184 | Lpg2239 | N/A | 3858 | This study |
| 185 | Lpg2248 | Lem21 | 2235 | [5] |
| 186 | Lpg2271 | N/A | 651 | This study |
| 187 | Lpg2298 | YlfA | 1275 | [16] |
| 188 | Lpg2300 | LegA3 | 1401 | [3] |
| 189 | Lpg2311 | Ceg28 | 3504 | [1] |
| 190 | Lpg2322 | LegA5 | 1923 | [3] |
| 191 | Lpg2327 | N/A | 891 | [4] |
| 192 | Lpg2328 | Lem22 | 384 | [5] |
| 193 | Lpg2344 | mavE | 627 | [1] |
| 194 | Lpg2351 | mavF | 921 | [1] |
| 195 | Lpg2359 | N/A | 444 | This study |
| 196 | Lpg2370 | N/A | 939 | This study |
| 197 | Lpg2372 | N/A | 1269 | This study |
| 198 | Lpg2382 | N/A | 1455 | This study |
| 199 | Lpg2391 | sdbC | 1305 | [1] |
| 200 | Lpg2392 | LegL6 | 885 | [3] |
| 201 | Lpg2400 | LegL7 | 1065 | [3] |
| 202 | Lpg2406 | Lem23 | 1098 | [5] |
| 203 | Lpg2407 | N/A | 306 | [4] |
| 204 | Lpg2409 | Ceg29 | 816 | Zusman, 2007 #477] |
| 205 | Lpg2410 | VpdA | 1998 | [14] |
| 206 | Lpg2411 | Lem24 | 828 | [5] |
| 207 | Lpg2416 | LegA1 | 1107 | [3] |
| 208 | Lpg2420 | N/A | 552 | [1] |
| 209 | Lpg2422 | Lem25 | 2622 | [5] |
| 210 | Lpg2424 | mavG | 1347 | [1] |
| 211 | Lpg2425 | mavH | 807 | [1] |
| 212 | Lpg2433 | Ceg30 | 1761 | [5] |
| 213 | Lpg2434 | N/A | 492 | This study |
| 214 | Lpg2443 | N/A | 558 | This study |
| 215 | Lpg2444 | mavI | 615 | [1] |
| 216 | Lpg2452 | LegA14 | 2766 | [3] |
| 217 | Lpg2456 | LegA15 | 1413 | [3] |
| 218 | Lpg2461 | N/A | 639 | This study |
| 219 | Lpg2464 | SidM | 1944 | [20] |
| 220 | Lpg2465 | SidD | 1521 | [7] |
| 221 | Lpg2490 | LepB | 3882 | [21] |
| 222 | Lpg2498 | mavJ | 933 | [1] |
| 223 | Lpg2504 | SidI | 2899 | [22] |
| 224 | Lpg2505 | N/A | 888 | This study |
| 225 | Lpg2508 | SdjA | 2421 | [19] |
| 226 | Lpg2509 | SdeD | 1195 | [7] |
| 227 | Lpg2510 | SdcA | 2727 | [7] |
| 228 | Lpg2511 | SidC | 2751 | [7] |
| 229 | Lpg2523 | Lem26 | 2340 | [5] |
| 230 | Lpg2525 | mavK | 1355 | [1] |
| 231 | Lpg2526 | mavL | 1368 | [1] |
| 232 | Lpg2527 | N/A | 1677 | [4] |
| 233 | Lpg2529 | Lem27 | 1719 | [5] |
| 234 | Lpg2538 | N/A | 1416 | This study |
| 235 | Lpg2539 | N/A | 408 | This study |
| 236 | Lpg2541 | N/A | 834 | [23] |
| 237 | Lpg2546 | N/A | 1410 | This study |
| 238 | Lpg2552 | N/A | 1668 | [1] |
| 239 | Lpg2555 | N/A | 855 | This study |
| 240 | Lpg2556 | LegK3 | 1386 | [3] |
| 241 | Lpg2577 | mavM | 759 | [1] |
| 242 | Lpg2584 | SidF | 2736 | [7] |
| 243 | lpg2588 | LegS1 | 1255 | [3] |
| 244 | Lpg2591 | Ceg33 | 495 | [2] |
| 245 | Lpg2603 | Lem28 | 1305 | [5] |
| 246 | Lpg2628 | N/A | 753 | This study |
| 247 | Lpg2637 | N/A | 1212 | This study |
| 248 | Lpg2638 | mavV | 1383 | [1] |
| 249 | Lpg2692 | N/A | 531 | This study |
| 250 | Lpg2694 | LegD1 | 858 | [3] |
| 251 | Lpg2718 | WipA | 1560 | [11] |
| 252 | Lpg2720 | LegN | 1029 | [3] |
| 253 | Lpg2744 | N/A | 1170 | [4] |
| 254 | Lpg2745 | N/A | 1968 | This study |
| 255 | Lpg2793 | LepA | 3453 | [21] |
| 256 | Lpg2804 | Lem29 | 1407 | [5] |
| 257 | Lpg2815 | mavN | 2052 | [1] |
| 258 | Lpg2826 | Ceg34 | 1734 | [5] |
| 259 | Lpg2828 | N/A | 1257 | This study |
| 260 | Lpg2829 | SidH | 6675 | [7] |
| 261 | Lpg2830 | LegU2 | 738 | [3] |
| 262 | Lpg2831 | VipD | 1863 | [6] |
| 263 | Lpg2832 | N/A | 1641 | This study |
| 264 | Lpg2844 | N/A | 1086 | This study |
| 265 | Lpg2862 | LegC8 | 1911 | [3] |
| 266 | Lpg2874 | N/A | 885 | [1] |
| 267 | Lpg2879 | mavO | 1752 | [1] |
| 268 | Lpg2884 | mavP | 738 | [1] |
| 269 | Lpg2885 | N/A | 555 | This study |
| 270 | Lpg2888 | N/A | 1914 | This study |
| 271 | Lpg2912 | N/A | 1488 | This study |
| 272 | Lpg2936 | N/A | 735 | This study |
| 273 | Lpg2975 | mavQ | 2616 | [1] |
| 274 | Lpg2999 | LegP | 798 | [3] |
| 275 | Lpg3000 | N/A | 1839 | This study |

References

1. Huang L, Boyd D, Amyot WM, Hempstead AD, Luo ZQ, et al. (2010) The E Block motif is associated with *Legionella pneumophila* translocated substrates. Cell Microbiol 13: 227-245.

2. Altman E, Segal G (2008) The response regulator CpxR directly regulates expression of several *Legionella pneumophila* icm/dot components as well as new translocated substrates. J Bacteriol 190: 1985-1996.

3. de Felipe KS, Pampou S, Jovanovic OS, Pericone CD, Ye SF, et al. (2005) Evidence for acquisition of Legionella type IV secretion substrates via interdomain horizontal gene transfer. J Bacteriol 187: 7716-7726.

4. Kubori T, Hyakutake A, Nagai H (2008) Legionella translocates an E3 ubiquitin ligase that has multiple U-boxes with distinct functions. Mol Microbiol 67: 1307-1319.

5. Burstein D, Zusman T, Degtyar E, Viner R, Segal G, et al. (2009) Genome-scale identification of *Legionella pneumophila* effectors using a machine learning approach. PLoS Pathog 5: e1000508.

6. Shohdy N, Efe JA, Emr SD, Shuman HA (2005) Pathogen effector protein screening in yeast identifies Legionella factors that interfere with membrane trafficking. Proc Natl Acad Sci U S A 102: 4866-4871.

7. Luo ZQ, Isberg RR (2004) Multiple substrates of the *Legionella pneumophila* Dot/Icm system identified by interbacterial protein transfer. Proc Natl Acad Sci U S A 101: 841-846.

8. Heidtman M, Chen EJ, Moy MY, Isberg RR (2008) Large-scale identification of *Legionella pneumophila* Dot/Icm substrates that modulate host cell vesicle trafficking pathways. Cell Microbiol.

9. Zusman T, Aloni G, Halperin E, Kotzer H, Degtyar E, et al. (2007) The response regulator PmrA is a major regulator of the icm/dot type IV secretion system in *Legionella pneumophila* and Coxiella burnetii. Mol Microbiol 63: 1508-1523.

10. Pan X, Luhrmann A, Satoh A, Laskowski-Arce MA, Roy CR (2008) Ankyrin repeat proteins comprise a diverse family of bacterial type IV effectors. Science 320: 1651-1654.

11. Ninio S, Zuckman-Cholon DM, Cambronne ED, Roy CR (2005) The Legionella IcmS-IcmW protein complex is important for Dot/Icm-mediated protein translocation. Mol Microbiol 55: 912-926.

12. Conover GM, Derre I, Vogel JP, Isberg RR (2003) The *Legionella pneumophila* LidA protein: a translocated substrate of the Dot/Icm system associated with maintenance of bacterial integrity. Mol Microbiol 48: 305-321.

13. Xu L, Shen X, Bryan A, Banga S, Swanson MS, et al. (2010) Inhibition of host vacuolar H+-ATPase activity by a *Legionella pneumophila* effector. PLoS Pathog 6: e1000822.

14. VanRheenen SM, Luo ZQ, O'Connor T, Isberg RR (2006) Members of a *Legionella pneumophila* family of proteins with ExoU (phospholipase A) active sites are translocated to target cells. Infect Immun 74: 3597-3606.

15. Ninio S, Celli J, Roy CR (2009) A *Legionella pneumophila* effector protein encoded in a region of genomic plasticity binds to Dot/Icm-modified vacuoles. PLoS Pathog 5: e1000278.

16. Campodonico EM, Chesnel L, Roy CR (2005) A yeast genetic system for the identification and characterization of substrate proteins transferred into host cells by the *Legionella pneumophila* Dot/Icm system. Mol Microbiol 56: 918-933.

17. Nagai H, Kagan JC, Zhu X, Kahn RA, Roy CR (2002) A bacterial guanine nucleotide exchange factor activates ARF on Legionella phagosomes. Science 295: 679-682.

18. Zusman T, Degtyar E, Segal G (2008) Identification of a hypervariable region containing new *Legionella pneumophila* Icm/Dot translocated substrates by using the conserved icmQ regulatory signature. Infect Immun 76: 4581-4591.

19. Liu Y, Luo ZQ (2007) The *Legionella pneumophila* effector SidJ is required for efficient recruitment of endoplasmic reticulum proteins to the bacterial phagosome. Infect Immun 75: 592-603.

20. Machner MP, Isberg RR (2006) Targeting of host Rab GTPase function by the intravacuolar pathogen *Legionella pneumophila*. Dev Cell 11: 47-56.

21. Chen J, de Felipe KS, Clarke M, Lu H, Anderson OR, et al. (2004) Legionella effectors that promote nonlytic release from protozoa. Science 303: 1358-1361.

22. Shen X, Banga S, Liu Y, Xu L, Gao P, et al. (2009) Targeting eEF1A by a *Legionella pneumophila* effector leads to inhibition of protein synthesis and induction of host stress response. Cell Microbiol 11: 911-926.

23. Ivanov SS, Charron G, Hang HC, Roy CR (2010) Lipidation by the host prenyltransferase machinery facilitates membrane localization of *Legionella pneumophila* effector proteins. J Biol Chem 285: 34686-34698.
