# Supplementary material for: Comprehensive Identification of Protein Substrates of the Dot/Icm Type IV Transporter of Legionella pneumophila
Source: PLoS One. 2011 Mar 9;6(3):e17638. doi: 10.1371/journal.pone.0017638 (PMC3052360; doi:10.1371/journal.pone.0017638)
Supplement: Table S5 — Expression of proteins fusions for candidates positive for translocation in the SidCΔC100 assay but negative in the β-lactamase reporter assay. (DOC) [file pone.0017638.s006.doc]

Table S5 Expression of proteins fusions for candidates positive for translocation in the SidC∆C100 assay but negative in the β-lactamase reporter assay

| Gene | Expression of the fusions | | Referenes |
| --- | --- | --- | --- |
|  | Cya fusion | β-lactamase fusion |  |
| lpg0107 | N/T | Yes | [1] |
| lpg0926 | Yes | No | [1] |
| lpg1152 | N/T | Yes | [1] |
| lpg1683 | N/T | Yes | [1] |
| lpg2344 | N/T | Yes | [1] |
| lpg2444 | N/T | Yes | [1] |
| lpg2525 | N/T | Yes | [1] |
| lpg2884 | N/T | Yes | [1] |

NT: Not tested

1. Huang L, Boyd D, Amyot WM, Hempstead AD, Luo ZQ, et al. (2010) The E Block motif is associated with *Legionella pneumophila* translocated substrates. Cell Microbiol 13: 227-245.
