# Supplementary material for: Comprehensive Identification of Protein Substrates of the Dot/Icm Type IV Transporter of Legionella pneumophila
Source: PLoS One. 2011 Mar 9;6(3):e17638. doi: 10.1371/journal.pone.0017638 (PMC3052360; doi:10.1371/journal.pone.0017638)
Supplement: Table S6 — Primers for construction of deletion mutants of Lpg2844. (DOC) [file pone.0017638.s007.doc]

Table S6 Primers for construction of deletion mutants of Lpg2844

| Primers | Target residues | Sequences |
| --- | --- | --- |
| Lpg2844C100 F | C100 Forward | 5'-CTGGGATCCAGCAGTGGCAGCGACAGTCAGTCCTCATCACCGCCTA  CAGACAGTAGCAGCAGTGGCAGC-3' |
| Lpg2844∆C100 F | ∆C100 Reverse | 5'-CTGGTCGACGCTACTGTCTGTAGGCGGTGATGAAGACTGGCTGTCGCTG  CCACTGCTGCTACTGTCTGT-3' |
